# Signs, beaches and bodies in pandemic times

**DOI:** 10.1177/1329878X20949980

**Published:** 2021-02

**Authors:** Marianne Clark

**Affiliations:** University of New South Wales, Australia

**Keywords:** affective atmosphere, beach, blue space, COVID-19, mundane governance, outdoor media environment, print signs

## Abstract

During the height of social distancing conditions in the first wave of the COVID-19 pandemic in Australia, the beaches of Sydney’s eastern suburbs became heavily regulated through prolific signage, physical barriers, and the presence of police and council staff. This essay explores the role of signage, as part of the outdoor media landscape, in contributing to the specific affective atmospheres in these extraordinary conditions and further demarcating Sydney’s beaches as exclusive spaces. Drawing on autoethnographic insights and visual imagery gathered during this time, I argue signs, as under explored forms of media, act as both mundane forms of governance and more-than-mundane contributors to the reconfiguration of affective and spatial relations.

On a sun-soaked Friday in March 2020, Sydneysiders flocked to Bondi Beach, enjoying the welcome vestiges of golden summer warmth after a season marked by extensive bushfires and the resulting environmental and emotional trauma. Quickly, the image of sunbathing bodies packed in close proximity became a viral image circulating with frenetic rapidity on social media and by news media around the world. While the sight of lounging bodies on Australian beaches is in no way exceptional, these were exceptional times. The world was in the midst of the global COVID-19 pandemic and this image captured the explicit flouting of social distancing rules, evoking ire on social media and disapproval from the global community.

The fallout was swift. The city’s iconic eastern suburbs beaches, including Bondi, Coogee, Bronte and Maroubra were closed the next day. Patrolled by council employees and decorated with fencing and extensive signage issuing directives about appropriate use, Sydney’s beaches, widely imagined as spaces of freedom and leisure, soon became deeply contested sites of potential risk and contagion. In the days and weeks that followed, and as some beaches opened for exercise, more signage appeared issued by local government authorities (LGAs). These signs ranged in style from cardboard placards encouraging social distancing and outlining permissible and prohibited activities, to large digital signs conveying clear and simple messages. ‘Beach Closed’ and ‘Exercise Only’ as shown in Figures 1 and 2.

**Figure 1.**
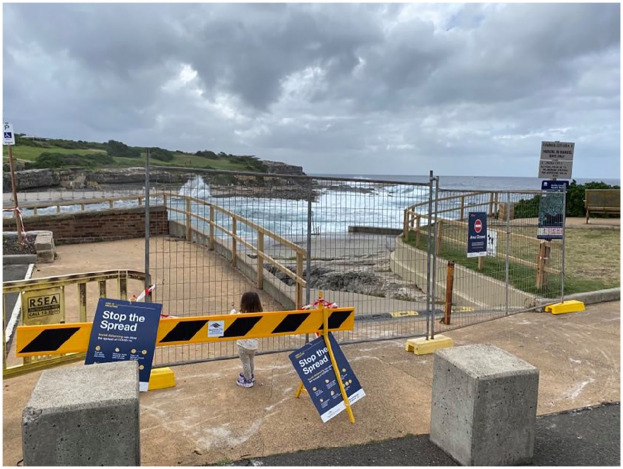
Physical barriers and signage prohibiting access to Clovelly Beach, Sydney.

**Figure 2.**
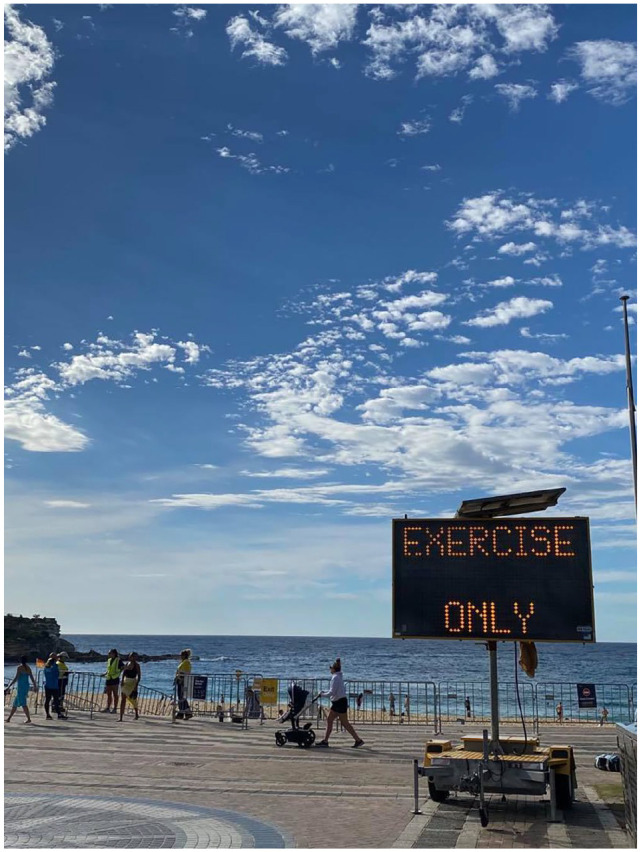
Digital sign looming over Coogee Beach, Sydney.

The signs prompted an array of responses, not all of which were predictable nor could be considered performances of compliance. Rather, people responded with bewilderment, indignance, longing, and, as this essay will explore, in creative and affective ways. While seemingly mundane, the signs carried exceptional messages that prompted visceral and corporeal responses and illuminated the complex entanglements of media, bodies and environments. Signs became implicated in the further hierarchisation of bodies that can and cannot access the beach, generated normative understandings about what forms of movement ‘count’, and illuminated the slippery and contested notions of ‘health’ within pandemic times.

In this essay, I employ a semi-autoethnographic approach (Ellis and Bochner, 2000) drawing on my personal location within the eastern Sydney beach-side suburb of Clovelly to explore how these signs, as everyday forms of media, became an intimate presence during the COVID-19 lockdown. I also draw from visual ethnography and provide personal images taken in this time to illustrate my arguments (Pink, 2013).

## Mundane governance in extraordinary times

As mundane urban objects, outdoor signage does not often garner attention as a significant form of media. Yet, urban geographer Kurt Iveson (2012) suggests that such signage, including everything from paid advertising billboards to graffiti, is integral to outdoor media landscapes. He defines these landscapes as media spaces that capture the attention of those passing through these environments as part of their everyday routines. Print signs often blend into the background of these spaces, becoming what we might consider banal. Yet, despite their mundanity, signs contribute to affective and aesthetic dimensions of our social landscapes and modulate movements of bodies. Woolgar and Neyland (2013), working at the intersections of science and technology studies and media and marketing studies, suggest signs are implicated in what they term ‘mundane governance’, or the ways in which governance in contemporary life is organised and enacted through everyday objects and technologies. Using airport signs as an example, they demonstrate the capacity of signs as a form of media that act not only as wayfinding technologies but as pervasive but non-objectionable forms of soft governance.

**Figure 3.**
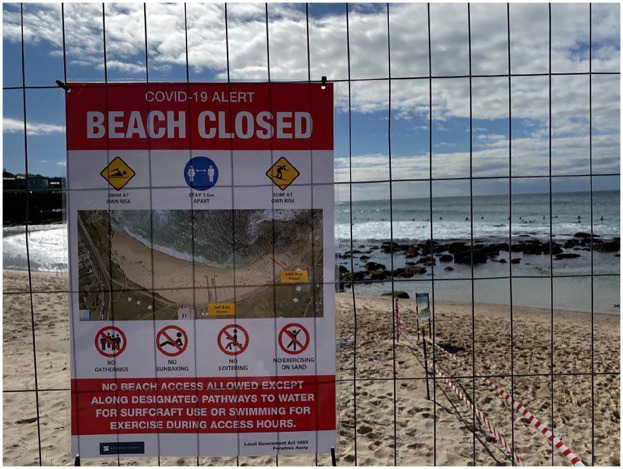
Sign indicating permissible beach activities at Bronte Beach, Sydney.

**Figure 4.**
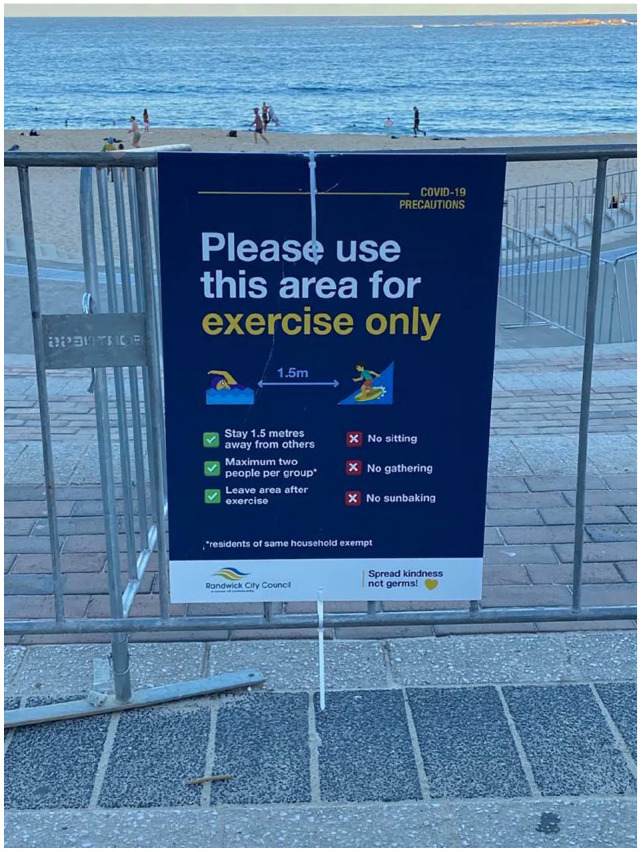
Sign indicating permissible beach usage at Coogee Beach, Sydney.

Indeed, signs deployed around the beaches of the eastern suburbs seemed intended to directly govern and regulate people’s movements in the interests of public health. While news media and LGA websites circulated updates on im/permissible beach use on their homepages and social media, the signage existed to remind (and confront) people as they encountered the beach and bordering outdoor spaces. The content and directives of the signs highlighted the risk of COVID-19 spread and outlined the steps needed to be taken to minimise such risks. This involved the practice of social distancing, and, in those signs specific to the beach, outlined exactly what activities were allowable in the water or on the sand, as shown in Figures 3 and 4.

The beach signs were deeply resonant with Foucault’s (1995) concept of disciplinary power as a form of governance over individual bodies. He argues power is deployed through techniques that target the body and specify how it is to be organised through time, space and movement. As a disciplining technology, signs indicated *where* bodies could move. In the case of Coogee and Maroubra beaches, people were allowed on the sand and in the water, as long as they were performing what was perceived to be a legitimate form of ‘exercise’ (no definition provided). At Bronte, Tamarama, and Bondi, when they finally opened after being firmly fenced off for weeks, only the water was accessible with swimming and surfing listed as permissible activities and exercise on the sand banned. For a short time, signage also indicated *when* people could be on the beach. Exercisers were allowed to use the beach from 6 am to 9 am; a 3-hour period where anxious, longing bodies bounded to the beach to perform bootcamp and burpees in a sandy, socially distanced, manner. These rules were enforced through signs with text and images, physical barriers and fencing, human patrol, police presence and auditory cues (the sound of an emergency alarm indicated the 9 am cut-off time).

While this use of signage resonates with the concept of mundane governance in some ways, there are important points of departure. In a rapidly shifting social and public health context marked by anxiety and worry, these signs were felt as anything but ordinary. Instead, this signage and the physical presence of traffic barriers and bright orange hazard tape confronted those who live by the beach – and for whom the sights, sounds and smell of the sea are an integral part of their day – with a visceral and jarring reminder of the dramatic disruption presented by COVID-19. Therefore, the signs emerged not only as vehicles of mundane governance or disciplinary power, but as vital, material forces – part of the affective atmosphere of everyday spaces entangled with moving bodies, environments and the intensified socio-emotional conditions of the global health pandemic.

Human geographer David Bissell elaborates on the ways things and objects become agentic through their relations with other human and non-human entities. In his examination of passenger experiences of commuting by train, Bissell (2010) observes that ‘the sociality of the railway carriage is tangled up as much with the agentic force of music players, signage, paper tickets, and seat backs, as with “individual” bodies’ (p. 286). In this example, mundane and seemingly trivial objects contribute to the overall ‘sociality’ of a space and lived experience, what Bissell refers to as an affective atmosphere. Affect here does not refer solely to emotion, but rather encompasses ‘how emotions, sensations, atmospheres and feelings arise out of relational encounters between objects, spaces and people’ (Spinney, 2015: 235). Therefore, the signs regarding exercise on the beach may be understood not merely as representations or vehicles for disciplinary power, but active participants in affective, material negotiations between human bodies, environments, geographies and social forces.

## ‘Exercise only’: signs as boundaries

In many ways, these signs redefined the beach from a place of relaxation, exercise, fresh air or engagement in leisure practices – all of which are usually associated with health and wellbeing – to a site of risk, contamination and fear of contagion. In so doing, these signs brought into focus the slipperiness and instability of the concept of ‘health’ in times of pandemic.

Signs also reorganised the social hierarchies of those who use and access the beach, producing beaches and blue spaces as even more socially, culturally and politically fraught. Within the Australian imaginary, the beach is largely storied as a place of egalitarian access, overlooking the complex ways in which social class matters (Ellison, 2014). Yet Australia’s beaches are the site of complex political tensions and performances of localism and racism: notably demonstrated through the Cronulla riots of 2005 (Barclay and West, 2006; Evers, 2008). Scholars have also noted how larger social, geographical and cultural forces act to constrain access to the beach for many, even pre-pandemic (Britton et al., 2019). Consequently, beach-going is deeply socially and materially contingent. With public beach parking lots closed and public transit largely considered a risky space during COVID-19, the possibilities for access were narrowed even further. Thus, the new signs restricting use were encountered almost soley by residents of neighbouring areas, rendering the noticing and responding to these signs an embodied performance of privilege.

The conditions for beach use prescribed by signs even further hierarchised those bodies ‘allowed’ on the beach and consequently, those with access to the associated mental and physical health benefits. In Bondi, Tamarama, and Bronte, only the water was accessible, and swimming and surfing were clearly listed as permissible activities. In Coogee and Maroubra, ‘Exercise Only’ was permitted on the sand as well as in the water. But what ‘counted’ as exercise on the sand was unclear, often extrapolated by users and those who ‘guarded’ these spaces to mean something that *looks* like conventional exercise such as running or performing bootcamp movements. On one occasion, my partner and I were allowed to remain on the beach with our 2-year-old daughter only because we were visibly performing such movements. At that same time, a ‘non-exercising’ woman and accompanying toddler were prompted off the beach, despite being the only other people present and a good 50 metres away from us. I recall the despair in her voice and the wail of the toddler.

In these ways, the beach became an even more exclusive space, with only those people wanting or able to perform such codified movements (and those with the time to do so) granted access, while others, including parents (most often mothers and female care providers) were directed away with their children, despite children’s bodies being in constant motion. The affective consequences of this were palpable, visible and audible – the physical and mental health benefits of the beach only accessible to very few in a time of social and emotional distress. Thus, there is much at stake for those bodies targeted by such signs, but the effects are not distributed or felt equally.

## Longing bodies

**Figure 5.**
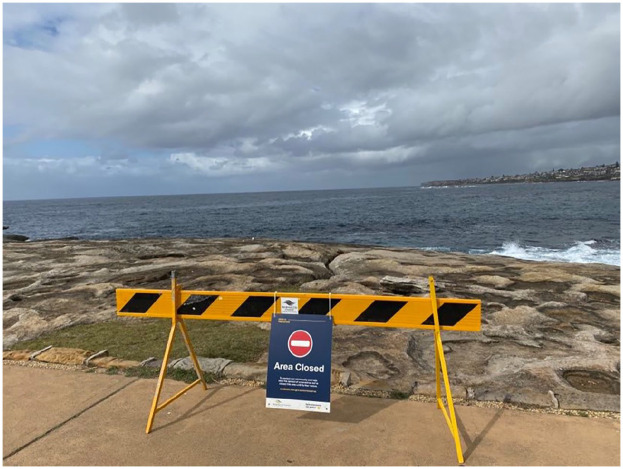
Signage and barrier near Clovelly Beach, Sydney.

**Figure 6.**
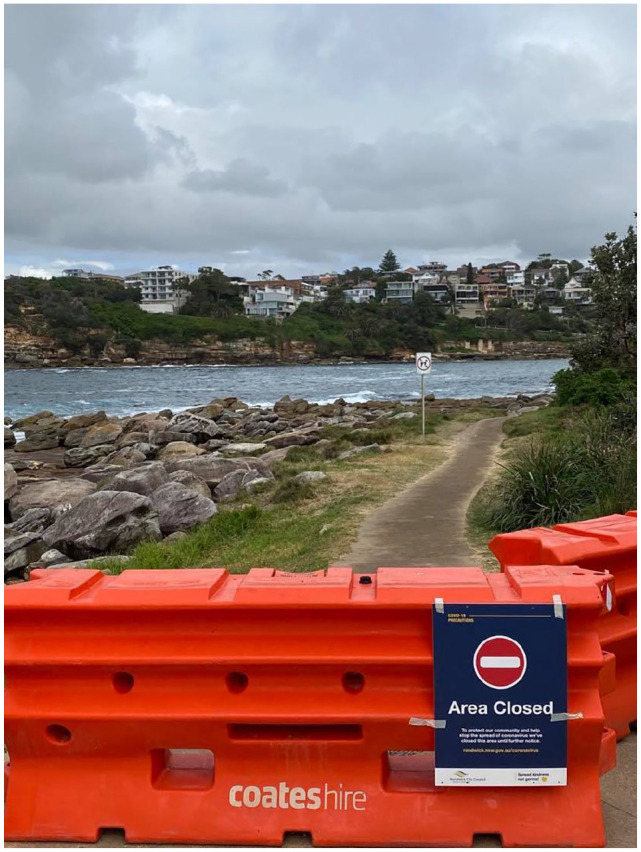
The glare against the beauty: barriers to Gordon’s Bay, Sydney.

What ensued from the narrowing conditions communicated through signage was a newfound and reconfigured relationship with the beach, the ocean and surrounding leisure spaces. These signs conveyed an extraordinary message to those bodies they encountered. ‘No access’. ‘Restricted’. These messages were jarring; a visceral response to unfamiliar social rules. So too was the sight of fluorescent plastic barriers against the backdrop of natural landscapes and the futility of the spindly construction barriers (see Figures 5 and 6) attempting to cordon off expanses of the seascape: as though this vast space could be contained and regulated. As though doing so would minimise our risk to the virus, itself proving to be uncontrollable.

While the signage implored people to do one thing, the body’s affective response was to create other ways to move, connect and make sense. In my neighbourhood, people negotiated new practices and routines involving the repurposing of spaces that did not involve the sandy shores of the beach nor immersion in the water but in which the ocean and the beach were still present. People sought out these spaces of the in-between, spilling into the unused Clovelly Beach parking lot to form an impromptu assemblage of children and bikes and scooters and parents moving with the scents and the sounds of the ocean. The exuberance of moving bodies in this strange re-assemblance was palpable to any passersby. Gordon’s Bay, an unpatrolled beach only accessible by pedestrians, saw an influx of intrepid open water swimmers, its waters more populated than in the preceding summer months. Moving bodies, if not always exercising bodies, were responding. They were also longing, exceeding the frames often reserved for them. Affective dimensions seeped through their movements, mingling with a sense of indignance and entitlement to blue spaces.

Therefore, while signs indeed acted as forms of mundane governance by regulating bodies and beach use, so too did they emerge as ‘more-than-mundane’ forces in the extraordinary conditions of the COVID-19 pandemic. Within these conditions, signs became entangled with humans, environments, geographies and social forces to reconfigure new social and spatial relations. In so doing, the instability of what counts as ‘healthy’ practice was rendered visible in new ways, as was the material production of inequitable access and the fragility of the Australian imaginary that celebrates the beach as a space of freedom.
